# Determination of Risk Factors and Treatment of Dry Eye Disease in Type 1 Diabetes Before Corneal Complications at Sindh Institute of Ophthalmology And Visual Sciences

**DOI:** 10.2174/1874364101711010355

**Published:** 2017-11-23

**Authors:** Shehnilla Shujaat, Muhammad Jawed, Shahzad Memon, Khalid Iqbal Talpur

**Affiliations:** Department of Ophthalmology, Sindh Institute of Ophthalmology and Visual Sciences Hyderabad, Hyderabad, Pakistan

**Keywords:** Dry Eye Disease, Risk factors, Association, Ocular surface, Corneal complication, Diabetes

## Abstract

**Objective:**

The objective of this study was to assess and determine the risk factors and treatment of dry eye disease in type 1 diabetes before any ocular surface or corneal complication occurs. This study was conducted at Sindh Institute of Ophthalmology And Visual Sciences, Hyderabad, Pakistan.

**Methodology:**

Subjects and methods for observational study were undertaken at the Department of Ophthalmology Sindh Institute Of Ophthalmology And Visual Sciences, Hyderabad, Pakistan. Hundred confirmed cases of type 1 diabetes were included in the study by non probability convenience sampling. Tear film breakup time and schrimer test were carried out to determine dry eye disease. Data was collected by self-prepared questionnaire and entered and analyzed by using Statistical Program for Social Sciences (SPSS, version 20.0). The frequencies and percentage were recorded and any associations with predisposing factors were statistically analyzed by t test.

**Results:**

Out of hundred patients, 71 (71%) were found to have dry eyes (P<0.001). The mean age of the subject in this study was 50.97 years (range 30-70 years). Old age was related to high risk of dry eye disease (P<0.001). There was no big difference in the incidence of dry eyes in males and females. Long duration was found to be related with increased occurrence of dry eyes (P<0.001). We found higher values for abnormal tear film break up time than schirmer test values leading to increased occurrence of evaporative dry eyes.

**Conclusion:**

There is marked increase in frequency/ risk of developing dry eye disease in type 1 diabetes patients. Therefore, it is recommended to have periodic ophthalmic examination for type 1 diabetic patients.

## 1 INTRODUCTION

Dry eye disease (DED) is a very common disease, associated with high osmolarity of the tear film and mild inflammation at the surface of eye [1]. The surface of eye consists of meibomian glands (the special sebaceous glands located at the eyelid margin to produce the outer lipid layer of the tear film), accessory lacrimal glands, conjunctiva, cornea, the main lacrimal gland, and the connection between all these structures collectively forms a proper functioning unit. Any one or all these components of ocular surface can be affected in dry eye disease [2]. Any stress to the surface of eye like change in environmental conditions, genetic factors, antigens, infection and endogenous stress is associated with the pathogenesis of dry eyes [3]. Pro inflammatory cytokines, matrix metalloproteinases and chemokines activate the autoreactive helper T cells which infiltrate the lacrimal gland and the surface of eye [4]. This results in a pathological inflammation and damage to the surface of eye [5]. DED is classified as “dry eye disease with decreased production of tears known as non-evaporative dry eye disease (aqueous deficient dry eye disorder)” and “dry eye disease associated with increased evaporative disorder of the tear film (hyper evaporative dry eye disorder)” [6]. This classification is very useful clinically. Hyper-evaporative dry eye disorder is usually caused by diseases of the meibomian glands, and mixed, aqueous deficient dry eye disorder and evaporative dry eye disorder forms are affecting 80% of patients [6, 7]. To prove these facts, new advanced procedures have evolved to diagnose and treat the dry eye disorder [8].

Dry eye is a disorder of tear film that results in ocular surface damage and ocular discomfort. Dry eye disorder is also called keratoconjunctivitis sicca (KCS). KCS is a Latin word and its meaning is dryness of cornea and conjunctiva [9]. Dry eye syndrome in which eyes does not produce sufficient tears is also known as aqueous deficient dry eye disorder. Hyper-evaporative dry eye disorder is usually associated with infections, infrequent blinking and other factors [10]. The estimated no of people experiencing the ocular discomfort due to dry eye disorder ranges from 25 till 30 million around the world [4, 7]. The occurrence of dry eye disease in diabetes is up to 54.3% [11]. In the United States, the reported frequency of dry eye disease was found to be 14.6% [12]. It is more common in middle aged women especially over 50 years of age due to autoimmune diseases [13]. The frequency of dry eye disorder is more prevalent in older age groups. The risk of the dry eye disease increases with old age, female gender, collagen vascular disease, antihistamines, postmenopausal estrogen treatment, refractive surgery of cornea, hepatitis c, androgen insufficiency, irradiation, hematopoietic stem cell transplantation, vitamin a deficiency, medications such as selective serotonin reuptake inhibitors, tricyclic antidepressants, diuretics, beta-blockers and diabetes mellitus [14].

The objective of this study was to assess and re-evaluate the risk factors and treatment with the association of dry eye disease in type 1 diabetes before any ocular surface or corneal complication occurs at Sindh Institute of Ophthalmology and Visual Sciences, Hyderabad, Pakistan.

## 2 MATERIAL AND METHODS

Cross sectional study was undertaken at the Department of Ophthalmology, Sindh Institute of Ophthalmology and Visual Sciences, Hyderabad. Duration of the study was six months and sample size was 100 patients and 200 eyes.

The prevalence of dry eyes in diabetic patients is 54.3%. Open- Epi epidemiological calculator was used for sample size calculation with confidence interval 95% and response distribution 50%. Non probability convenience sampling technique was used.

The Tear Film Break-Up Time (TFBUT) is an indicator of the tear film stability over the ocular surface. It is a very important diagnostic test. In this test, fluorescein drops are installed without any topical anaesthesia and checked with slit lamp with a cobalt blue filter. After one complete blink of eye, the time is noted for the first tear film break- up. It is taken as normal between 20 and 30 seconds. TBUT values are considered as abnormal, when these are 0 to 10 seconds. Another very important test is Schrimer test. It is checked with a special study to measure the secretions of the lacrimal gland. In the Schirmer test, Whatman filter paper number 41 is used. Paper (35 × 5 mm) is placed in the lower fornices, at the junction of medial two third and temporal one third of the lower eyelid. The patient has to keep his eyes closed for 5 minutes. Following this, wetting of the paper strip is measured and values are reduced in non-evaporative or aqueous-deficient dry eye. Values are considered pathological when these are 5mm or less.

### 2.1 Data Collection

A total of 100 patients, of 30 years of age and above diagnosed as type 1 diabetics attending the outpatient department for various ocular problems were assessed for dry eyes. Patient’s ages were recorded at the time of diagnosis of type 1 diabetes mellitus (Table **1**). On the basis of the history and record, they were diagnosed as the patients of type 1 diabetes mellitus. The initial examination consisted of slit lamp bi-microscopy; the patients were selected from general OPD of Sindh Institute of Ophthalmology and Visual Sciences. Clinical data regarding patient’s age, gender, duration of diabetes, insulin dependency, controlled or uncontrolled insulin metabolism and history of other diseases were recorded by reviewing the patient’s medical record and direct interviews. Patients diagnosed with type 1 diabetes were further assessed for dry eyes. Patients went through comprehensive ophthalmic examinations by author. TBUT and schrimer test were done on each patient and severity was recorded as 1.mild 2.moderate and 3.severe. Data was entered in the specific proforma. Dry eyes disorder was diagnosed on the basis of the complaint of foreign body sensation, itching, redness and mucoid discharge [15]. The disorder was confirmed by TBUT and schrimer test [16]. Ocular surface was accessed with the help of slit lamp. Informed consent was obtained from all subjects. In TBUT test, appearance of dry spot within 10 sec with local anesthesia was considered abnormal. In schrimer test, wetting of paper within 06 mm after 5 minutes with local anesthesia was considered abnormal.

### 2.2 Data Analysis

Observational statistics were analyzed by SPSS version 20.0. Break up time of tear film within 10 sec with local anesthesia was observed to be abnormal. In schrimer test, wetting of paper within 06 mm after 5 minutes with local anesthesia was considered as abnormal. P-value <0.05 was considered significant. The statistics tests will be applied as appropriate.

## 3 RESULTS

Out of 100 patients, minimum duration was 6 years and maximum duration was 30 years with mean duration and standard deviation being16.24 years and 5.091, respectively (Table **2**).

Break up time of tear film was seen in the right eyes of 100 patients of type 1 diabetes mellitus. Break up time of tear film was 0 seconds and the maximum was 20 seconds. Mean tear film break up time was 7.20 seconds and standard deviation was 5.512.

Break up time of tear film was seen in the left eyes of 100 patients. Minimum break up time of tear film was 0s and maximum was 19s. Mean tear break up time of left eyes was 7.68 and standard deviation was 5.128 (Table **3**).

Schirmer test was done on the right eyes of 100 patients. Minimum schirmer test value was 0 mm and maximum was 30 mm. Mean schirmer test value was 12.21 and standard deviation was 6.812 (Table **4**).

Schirmer test was done in the left eyes from 100 patients of type 1 diabetes. Minimum value was 0 mm and maximum value was 28 mm. Mean schirmer test value of left eyes was 11.84 and standard deviation was 7.212 (Table **5**).

Break up time of tear film was estimated in both the eyes of 100 patients. We found mild DED in 28% of the patients, moderate DED in 25% and severe DED in 18% of the patients. We found DED in 71% of patients suffering from diabetes, so in our hospital, we found enormous percentage of patients suffering from DED. This indicates that the high prevalence of evaporative DED in our population is due to ocular surface diseases (Table **6-10**).

## 4 DISCUSSION

Diabetes mellitus is known as a greatly prevalent systemic illness affecting a large population [2, 11, 17]. Diabetes mellitus is strongly associated with DED [5]. There are only few studies conducted on the association of type 1 diabetes and dry eye disease. As this is a very commonly occurring ocular problem and can aggravate much ocular morbidity, DED should be examined in every patient attending outpatient department to reduce the dangerous complications of this disease leading to blindness. DED is again a highly prevalent disease among several common ocular problems. It can initiate and magnify many ocular diseases leading to several corneal complications which then eventually leads to blindness. DED is divided into evaporative and non-evaporative dry eye disorder. Evaporative DED is due to ocular surface and meibomian gland diseases. Non-evaporative DED is common in females due to autoimmune diseases and old age patients due to age related dysfunction of lacrimal gland. So we decided to highlight this very important ocular morbidity. Examination and treatment of dry eye disease are not only meant to determine dry eye disease but to reduce almost all ocular morbidities leading to severe corneal complications. Several studies were conducted to see the occurrence of DED in type 1 diabetes and its relation with duration of diabetes mellitus, gender and old age [18, 19]. There are many tests to examine DED, but tear film break up time and schirmer test are the most significant among all other available investigations [20]. Evaporative DED is reflected by abnormal tear film break up time values and non-evaporative by abnormal schirmer test results. Evaporative type is the most common type of DED. In our study, total 100 patients of type 1 diabetes were examined, out of which 52 (52%) were males and 48 (48%) were females. In our patients, the minimum age was 32 years, the maximum age was 70 years, and the mean age was 50.97 years. Two tests were conducted on our patients; these were, the break up time of tear film and schirmer test and then their results were analyzed to see that how much percentage of diabetic patients were suffering from DED. Analysis showed that minimum break up time of tear film was 0 seconds, maximum was 19 seconds and mean tear film break up time was 7.68 seconds in the left eyes. Results of breakup time of tear film showed that majority of patients had abnormal values. It means many patients were suffering from DED. These patients were also having ocular surface diseases and bad eye lid hygiene. So eyelid hygiene should be considered in etiology of ocular surface problems, which then lead to dry eye disease. Second test done on these patients was schirmer test showing the basal secretions of lacrimal glands. If the basal secretions are disturbed, then patient develop non-evaporative DED. Non-evaporative DED is common in old age due to age related dysfunction of lacrimal gland specially in females due to autoimmune diseases. Diabetic patients should be examined for ocular surface diseases along with routine fundus examination [21]. In a study conducted by Masoud Raza manaviat, there was no significant association between gender and frequency of DED. He reported 66.7% DED in 65-85 years old age patients and lower percentage of patients suffering from DED in 27-41 years old age group. Duration of diabetes mellitus is again a very important factor in the initiation of DED. It should be considered always in pathogenesis and severity of DED. In our study, the minimum duration of type 1 diabetes was 6 years, maximum duration was 30 years and mean duration was 16.24 years. Prevalence and severity of dry eye disease was observed to be increasing with increasing duration of diabetes mellitus. When the association of DED and duration was analyzed, P value was significant (P<0.001). In our study there were 48 females, comprising almost half of our study population and representing almost 50% of our study. In our study, 76.9% of males and 64.5% of females were suffering from dry eye disease. We did not find increased prevalence of DED in females as compared to males. There was almost equal prevalence of DED in males and females. So gender was not a risk factor in our study. In one cohort study conducted on 3722 subjects, with age ranging from 48-91 year, the overall prevalence was 14.4% in diabetic patients, 8.4% in subjects younger than 60 years and 19% in subjects older than 80 years. So there was strong association of dry eye disease with old age. Saifart et al. compared 92 type 1 and type 2 diabetics, aged from 7-69 years with a normal healthy group (controls, comparable in number, aged and gender). They found dry eye disease in 52.8% diabetics. In another study, 140 patients, with age ranging from 20-93 year, suffering from DED disease were assessed. They found the higher prevalence of dry eye syndrome in those patients, with 80% females being affected from DED. In another study conducted by kim EC in 2010, 100 patients of type two diabetes were examined. They compared diabetics with normal subjects and found significant lower values of tear film break up time in diabetic patients. In another study conducted on 2414 subjects, 322 patients developed DED over a period of 5 years, in which the incidence of dry eyes syndrome was significantly associated with age and diabetes mellitus. DED was assessed in 30 Pakistani patients. Out of 30 patients, 20 were males and 10 females ranged from under 20 to over 60 years. The visual acuity of those patients at the time of presentation was 6/60(20/200) or decreased in 21(70%) patients and 6/18(20/60) or better in nine (30%). Fifteen (50%) patients were blind and another six (20%) had very poor prognosis. Only nine (30%) patients had visual prognosis [22].

## CONCLUSION

In our study, old age and duration of diabetes mellitus were risk factors. The prevalence of dry eye disease was more in old age and in patients with longer duration of diabetes mellitus. The results of our study were compared between males and females. The prevalence of dry eye disease was same in males and females. We found more abnormal values in tear film break up time and less abnormal values in schirmer test results. So in our study, the prevalence of evaporative dry eye disease was more than non-evaporative dry eye disease.

## Figures and Tables

**Table 1:** Descriptive statistics of duration (years) of diabetes mellitus type1.

	N	Minimum	Maximum	Mean	Std. Deviation
DURATION OF DIABETES MELLITUS TYPE 1	100	6y	30y	16.24	5.091

**Table 2:** Range of tear film break up time (TBUT) of right eye (R/E).

	N	Minimum	Maximum	Mean	Std. Deviation
TBUT of R/E	100	0s	20s	7.2	5.512

**Table 3:** Range of tear film break up time of left eye.

	N	Minimum	Maximum	Mean	Std. Deviation
TBUT of L/E	100	0s	19s	7.68	5.128

**Table 4:** Range of schirmer of right eye.

	N	Minimum	Maximum	Mean	Std. Deviation
SCHIRMER test of R/E	100	0mm	30mm	12.21	6.812

**Table 5:** Range of schirmer test of left eye.

	N	Minimum	Maximum	Mean	Std. Deviation
SCHIRMER test of L/E	100	0mm	28mm	11.84	7.212

**Table 6:** Results of mild, moderate and severe evaporative dry eye disease in both eyes.

**Both Eyes**	**Frequency**	**Percent (%)**	**Valid Percent**	**Cumulative Percent**
**Mild**	28	28	28	28
**Moderate**	25	25	25	53
**Severe**	18	18	18	71
**Normal**	29	29	29	100
**Total**	100	100.00%	100.00%	

**Table 7:** Correlation OF AGE and TBUT B/E.

		AGE	TBUTB/E
AGE	Pearson Correlation	1	-.223*
	Sig. (2-tailed)		.026
	N	100	100
TBUT B/E	Pearson Correlation	-.223*	1
	Sig. (2-tailed)	.026	
	N	100	100
*. Correlation is significant at the 0.05 level (2-tailed).			

This table shows that old age is strongly associated with DED in the Diabetes. DED in the diabetes increases with the old age.

**Table 8:** Correlation of DURATION and TBUT B/E.

		DURATION	TBUT B/E
DURATION	Pearson Correlation	1	-.295**
	Sig. (2-tailed)		.003
	N	100	100
TBUT B/E	Pearson Correlation	-.295**	1
	Sig. (2-tailed)	.003	
	N	100	100
**. Correlation is significant at the 0.01 level (2-tailed).			

This table shows that the duration of the diabetes is strongly associated with DED in diabetes. DED in diabetes increases with the longer duration of diabetes.

**Table 9:** Comparison of the TBUT B/E and SCIRMMER B/E.

		TBUT B/E	SCIRMMER B/E
TBUT B/E	Pearson Correlation	1	.371**
	Sig. (2-tailed)		.000
	N	100	100
SCIRMMER B/E	Pearson Correlation	.371**	1
	Sig. (2-tailed)	.000	
	N	100	100
**. Correlation is significant at the 0.01 level (2-tailed).			

This table shows the comparison of two tests performed in the DED, these are TBUT B/E and SCIRMMER B/E. In this table, when the TBUT increases, the Schirmer value also increases.

**Table 10:** Comparison of DED in diabetes in previous study and our study.

**Manviat study**	**Our study**
**DED were 54.3%**	**DED were 71%**

This table shows that DED in diabetes was 54.3% in Manviat study and 71% in or study.
